# Effects of Bisphosphonates on Bone Micro‐Architecture of Children With Duchenne Muscular Dystrophy: A Prospective Comparative Study

**DOI:** 10.1002/jcsm.70227

**Published:** 2026-02-26

**Authors:** Songqi Wang, Yi Dai, Lingyang Meng, Yi Zhang, Lei Sun, Yanye Wang, Ou Wang, Yan Jiang, Weibo Xia, Xiaoping Xing, Wei Yu, Mei Li

**Affiliations:** ^1^ Department of Endocrinology, National Health Commission Key Laboratory of Endocrinology Peking Union Medical College Hospital, Chinese Academy of Medical Science and Peking Union Medical College Beijing China; ^2^ Department of Neurology Peking Union Medical College Hospital, Chinese Academy of Medical Science and Peking Union Medical College Beijing China; ^3^ Department of Radiology Peking Union Medical College Hospital, Chinese Academy of Medical Science and Peking Union Medical College Beijing China

**Keywords:** alendronate, Duchenne muscular dystrophy, osteoporosis, zoledronic acid

## Abstract

**Background:**

Duchenne muscular dystrophy (DMD) is an X‐linked recessive disorder that affects dystrophin production, characterized by progressive neuromuscular dysfunction, often accompanied by osteoporosis. We prospectively evaluate the effects of bisphosphonates on bone micro‐architecture reflected by trabecular bone score (TBS) of patients with DMD.

**Methods:**

A total of 72 male children or adolescents with DMD were included, with a mean age of 9.5 ± 1.8 years. They were divided into bisphosphonate treatment groups and control group based on areal bone mineral density (aBMD) and history of fragility fractures. Patients in bisphosphonate treatment groups randomly received intravenous infusion of 5 mg zoledronic acid (ZOL) annually or oral 70 mg alendronate weekly for three years. All patients took calcium 600 mg plus 125 IU vitamin D daily and calcitriol 0.25 μg every other day. TBS at the lumbar spine (LS) and aBMD at the LS, femoral neck (FN) and total hip (TH) were measured annually by dual‐energy X‐ray absorptiometry. Serum levels of β‐isomerized carboxy‐telopeptide of type I collagen and alkaline phosphatase were measured annually during the follow‐up.

**Results:**

A total of 25 (86.2%), 26 (92.9%) and 13 (86.7%) patients in the ZOL, alendronate and control groups completed the study. After 3 years, TBS Z‐score increased from baseline by 1.13 (*p* < 0.01), 0.68 (*p* < 0.01) and 0.26 (*p* > 0.05) in the ZOL, alendronate and control groups, respectively. The mean increase in TBS Z‐score from baseline was significantly greater in both bisphosphonate treatment groups compared to the control group (*p* < 0.05). No significant difference was found between the ZOL and alendronate groups. LS, FN and TH aBMD increased by 35.8%, 23.7% and 34.5% in the ZOL group (all *p* < 0.01 vs. baseline and control group) and by 21.5%, 29.3% and 25.0% in the alendronate group (all *p* < 0.05 vs. baseline and control group). LS and FN aBMD Z‐scores increased by 1.56 and 1.63 in the ZOL group (all *p* < 0.01 vs. baseline), by 1.32 and 1.48 in the alendronate group (all *p* < 0.05 vs. baseline). Bisphosphonates demonstrated a favourable safety profile during the study period.

**Conclusion:**

This relatively long‐term study confirms that zoledronic acid and alendronate are beneficial to improve micro‐architecture reflected by TBS and aBMD of children or adolescents with DMD.

## 1 Introduction

Duchenne muscular dystrophy (DMD) is an extremely rare, X‐linked neuromuscular disease, affecting approximately 1 in 4000–6000 male births [1]. DMD is caused by pathogenic variants in the *DMD* gene that result in the absence of functional dystrophin in muscle, which leads to progressive impaired motor function, loss of ambulation and impaired cardiorespiratory function [2, 3]. Radical treatment strategies are still lacking in DMD and glucocorticoids (GCs) are the main therapeutic drugs, which can effectively delay disease progression and improve the quality of life in DMD patients [4, 5].

GCs are known to reduce bone formation and increase bone resorption, resulting in early‐onset osteoporosis and marked deterioration of the bone microstructure [6]. Reduced activity and immobilization of the body can lead to accelerated bone loss [7]. Immobilization and GC therapy can induce osteoporosis and consequently substantially increase the risk of fragility fractures. These complications lead to impaired quality of life and can even be life‐threatening for DMD patients [8, 9].

Bisphosphonates are effective agents for osteoporosis by inhibiting osteoclast‐mediated bone resorption [10]. Bisphosphonates include oral medications (e.g., alendronate) and intravenous formulations (e.g., zoledronic acid, ZOL) [10]. Intravenous bisphosphonates exhibit high bioavailability and are administered annually, with minimal gastrointestinal side effects. This profile renders them particularly suitable for patients with gastrointestinal dysfunction. However, acute‐phase reactions, such as fever, are commonly observed following the initial infusion. In contrast, oral bisphosphonates require weekly administration and are not associated with acute‐phase reactions. Nevertheless, they are characterized by lower bioavailability and may induce gastrointestinal adverse effects, including gastroesophageal reflux and heartburn [11]. Previously study proved that bisphosphonates could significantly inhibit bone resorption, increase areal bone mineral density (aBMD) and reduce the risk of fractures in patients with GCs induced osteoporosis (GIOP) [12, 13]. However, it is noteworthy that patients with GIOP exhibit significant damage to bone microstructure and may experience fragility fractures even when the aBMD is not very low and aBMD cannot fully reflect the risk of fracture in patients with GIOP [14, 15].

Recently, the trabecular bone score (TBS), derived from grey‐level variations in lumbar dual‐energy X‐ray absorptiometry (DXA) images, can reflect three‐dimensional trabecular structural characteristics and become a new imaging indicator that effectively reflects bone microstructure [16]. The International Society for Clinical Densitometry (ISCD) recommends TBS as a supplementary assessment tool for GIOP patients and highlights its clinical advantages in detecting early microstructural damage in patients with normal aBMD and predicting vertebral fracture risk [16]. However, the changes in TBS in DMD patients are not clear and there are no research reports about whether it can reflect the efficacy of bisphosphonates in DMD patients.

Therefore, this prospective study aims to evaluate the effects of bisphosphonate therapy on changes in TBS and aBMD in patients with DMD and to compare the efficacy differences between oral and intravenous bisphosphonates.

## 2 Subjects and Methods

### 2.1 Study Design

This was a 3‐year, prospective, open‐label study. The study was approved by the Scientific Ethics Committee of Peking Union Medical College Hospital (PUMCH, 1‐24PJ1494). The legal guardians of all patients provided written informed consent before they participated in this study.

### 2.2 Subjects and Treatment

The patients were recruited from the Departments of Endocrinology and Neurology at PUMCH from 2012 to 2024. Children and adolescents were eligible if they fulfilled the following inclusion criteria: diagnosed with DMD, which was confirmed by carrying *DMD* gene mutations or absence of dystrophin on muscle biopsy. The exclusion criteria were as follows: other muscular dystrophy, such as suspected Becker muscular dystrophy; a history of other metabolic bone diseases, such as hyperparathyroidism, rickets and osteogenesis imperfecta; with malignant tumours; receiving treatments other than GCs that are known to affect bone metabolism; with severe hepatic or renal impairment (estimated glomerular filtration rate below 35 mL/min); and allergic to bisphosphonates.

Enrolled patients were divided into two cohorts: those who met either of the following criteria entered the bisphosphonate treatment cohort: (1) aBMD Z‐scores less than −2.0, or (2) a history of nontraumatic fractures of the spine, hip, wrist, or humerus. Participants with BMD Z‐scores greater than −2.0 and without a history of nontraumatic fractures entered the control cohort. Patients in the bisphosphonate treatment cohort were further randomized and assigned to either the ZOL group, receiving an annual intravenous infusion of 5 mg zoledronic acid (Aclasta, Novartis Pharmaceuticals, Switzerland), or the alendronate group, receiving 70 mg alendronate orally once weekly (Fosamax, Merck, UK). All patients in the three groups (the two bisphosphonate treatment groups and the control group) received the same supplementation of daily calcium (600 mg) and vitamin D (125 IU, Caltrate D; Wyeth Pharmaceuticals), along with oral calcitriol (0.25 μg, Rocaltrol; R.P. Scherer GmbH & Co. KG, Germany) administered on alternate days. This foundational regimen was maintained for 3 years.

### 2.3 Endpoints of the Study

The primary endpoints were the changes in TBS and TBS Z‐score at the lumbar spine (LS), aBMD at the LS, femoral neck (FN), trochanter (TR) and total hip (TH) after 3 years. The secondary endpoints were the changes in bone turnover biomarkers (BTMs), including β‐isomerized carboxy‐telopeptide of type I collagen (β‐CTX, bone resorption biomarker) and alkaline phosphatase (ALP, bone formation biomarker).

Serum levels of other bone metabolic biochemical indicators, including calcium (Ca), phosphate (P), parathyroid hormone (PTH) and 25‐hydroxyvitamin D (25(OH)D) were evaluated during the treatment. Safety of the treatment was assessed by clinical records and laboratory tests and the adverse events (AEs) were evaluated during each follow‐up visit.

### 2.4 Measurement of aBMD and TBS

The aBMD at the LS, FN, TR and TH was measured using DXA (Lunar Prodigy Advance, GE Healthcare, USA) at baseline, 12, 24 and 36 months of the treatment. All scans of patients were performed on the same DXA machine and the phantom was tested daily using the DXA device for calibration and quality checks. Vertebrae with severe deformities were excluded from the analysis of aBMD measurement results. The coefficients of variation for DXA measurements were 1.1% and 1.7% at LS and FN, respectively. Based on the aBMD reference data of normal children in China, the Z‐scores of aBMD at LS and FN were calculated [17]. The TBS values were derived from the vertebrae L1‐L4 DXA scans using TBS iNsight v2.1 software (Med‐Imaps, Merignac, France), with analysis performed by professional radiologists. In addition, patients with a body mass index (BMI) below 15 or above 37 kg/m^2^ were excluded as TBS analysis is not recommended in these patients. Lumbar TBS was calculated as the mean value of individual measurements for vertebrae L1‐L4. Age‐, sex‐, height‐ and weight‐adjusted Z‐scores of TBS were calculated using the reference data of healthy children and adolescents obtained from the same DXA devices [18].

### 2.5 Measurement of Serum Biochemical Indexes

Fasting blood samples were collected from 08:00 to 10:00 in the morning at baseline, 12, 24 and 36 months of the treatment. Serum levels of β‐CTX, 25(OH)D and PTH were measured using an automated electrochemiluminescence system (E170, Roche Diagnostics, Switzerland). Serum levels of ALP, alanine aminotransferase (ALT), Ca, P, fast blood glucose (FBG) and creatinine (Cr) were measured by automated analysers (ADVIA 1800, Siemens, Germany), which were uniformly measured in the central clinical laboratory of PUMCH.

### 2.6 Assessment of Bone Fractures

The information on bone fracture at baseline was obtained during the collection of medical history, including frequency and site of fracture and external trauma conditions, and the vertebral compression fractures were confirmed by X‐ray imaging by a radiologist. New fractures during the treatment were self‐reported by the patient and their guardian and confirmed by X‐ray examination. During the treatment period, anteroposterior and lateral X‐ray imaging of the thoracolumbar spine were performed annually to detect new vertebral compression fractures, which were graded as mild (Grade 1), moderate (Grade 2) or severe (Grade 3) according to the Genant semiquantitative method [19].

### 2.7 Safety Assessment

Biochemical indicators of liver and kidney function were measured at baseline, 12, 24 and 36 months of the treatment. In addition to routine reporting of all AEs, the patients' symptoms and signs were collected in detail during each visit. Serious AEs were closely monitored, including severe biochemical abnormalities, hospitalizations, osteonecrosis of the jaw, atypical fracture, atrial fibrillation and even death.

### 2.8 Statistical Analysis

The Kolmogorov–Smirnov test was performed to assess normality of continuous variables. Normally distributed data (including aBMD, β‐CTX, ALP, Ca, P, 25(OH)D, PTH) were presented as mean ± standard deviation (SD). Non‐normally distributed variables (GC treatment time) were expressed as medians and interquartile ranges (IQRs). Categorical data including fractures history and the incidence of AEs were expressed as numbers and percentages (%). Paired t tests were conducted to compare the differences in aBMD, TBS and their Z‐score, BTMs, Ca, P, 25(OH)D, PTH, ALT, Cr and FBG from baseline. Independent t‐test and one‐way ANOVA were used to compare the differences in continuous variables between various groups.

Statistical analyses were performed using SPSS software (version 27.0; SPSS Inc., Chicago, IL, USA) and statistical significance was considered when *p* value was less than 0.05. Graphs were produced using GraphPad Prism software version 10.1.2 (GraphPad Software, La Jolla, CA, USA).

## 3 Results

### 3.1 Characteristics of DMD Patients at Baseline

A total of 72 DMD patients with an average age of 9.5 ± 1.8 years were included in this study. A total of 68 patients (94.4%) with DMD were receiving prednisolone or equivalent GC therapy. The proportion of patients undergoing GC therapy showed no statistically significant difference among the three groups (*p* = 0.641). The median duration of GC therapy was 24 months in both the ZOL and alendronate groups and 20 months in the control group, with no significant intergroup difference (*p* = 0.430). The baseline prednisolone or equivalent GCs dose was 0.52 ± 0.25 mg/kg/day in the ZOL group, 0.46 ± 0.37 mg/kg/day in the alendronate group and 0.59 ± 0.28 mg/kg/day in the control group, with no statistically significant difference among the three groups (*p* = 0.541). Additionally, no significant baseline differences were observed among the three groups in terms of aBMD and aBMD Z‐score, serum levels of β‐CTX, ALP, Ca, P, PTH, 25(OH)D, Cr and FBG (*p* > 0.05 for all; Table 1). Serum levels of ALT at baseline in all three groups were significantly higher than the normal range, with serum Cr levels significantly lower than the normal range (Table 1).

**TABLE 1:** Characteristics of the patients with DMD at baseline.

	ZOL group	ALN group	Control group	*p*	Reference
n	29	28	15		
Age (years), mean (SD)	10.40 ± 2.01**	9.23 ± 1.48	8.71 ± 1.37	**0.005**	
Height (cm), mean (SD)	135.79 ± 11.46 **	128.52 ± 12.73	125.13 ± 9.73	**0.016**	
Weight (kg), mean (SD)	37.83 ± 11.24 **	32.54 ± 8.34	29.86 ± 5.89	**0.025**	
BMI (kg/m^2^), mean (SD)	19.87 ± 3.85	17.65 ± 2.27	18.43 ± 3.13	0.113	
Height *Z*‐score, mean (SD)	−0.31 ± 1.10	−0.36 ± 1.59	−0.20 ± 1.00	0.948	
Weight *Z*‐score, mean (SD)	0.73 ± 1.14	0.36 ± 1.04	0.50 ± 0.63	0.532	
Patients receiving GC therapy, *n* (%)	28 (96.6%)	27 (96.4%)	13 (86.7%)	0.641	
Duration of GC therapy (month), median (IQR)	24 (16, 32)	24 (15.5, 34)	20 (11.5, 30.5)	0.430	
Current GC dose (mg/kg/d), mean (SD)	0.52 ± 0.25	0.46 ± 0.37	0.59 ± 0.28	0.541	
Ambulation with aids, *n* (%)	10 (34.48)	8 (28.57)	2 (13.33)	0.057	
Fragility history, *n* (%)	9 (31.03)	8 (32.14)	0 (0)	**0.042**	
Ca (mmol/L)	2.38 ± 0.05	2.41 ± 0.07	2.40 ± 0.07	0.470	2.13 ~ 2.70
P (mmol/L)	1.59 ± 0.14	1.64 ± 0.16	1.70 ± 0.20	0.143	1.29 ~ 1.94
ALP (U/L)	98.80 ± 29.37	85.55 ± 31.49	75.33 ± 34.61	0.474	58 ~ 400
PTH (pg/mL)	26.75 ± 14.38	20.72 ± 4.99	22.69 ± 7.48	0.422	15.0 ~ 65.0
*β*‐CTX (ng/mL)	0.67 ± 0.30	0.60 ± 0.35	0.64 ± 0.35	0.765	0.21 ~ 0.44
25(OH)D (ng/mL)	16.05 ± 5.39	16.85 ± 3.88	17.14 ± 6.81	0.745	30.0 ~ 50.0
ALT (U/L)	185.85 ± 92.29	269.82 ± 145.13	310.72 ± 109.82	**0.005**	5 ~ 90
Cr (umol/L)	20.85 ± 6.27	22.78 ± 6.79	21.81 ± 5.65	0.572	59 ~ 104
FBG (mmol/L)	5.03 ± 1.10	4.89 ± 0.62	4.85 ± 0.61	0.827	3.9 ~ 6.1
TC (mmol/L)	4.08 ± 0.50	4.73 ± 0.23	4.54 ± 0.39	0.276	< 5.18
TG (mmol/L)	1.47 ± 0.12	1.40 ± 0.53	1.45 ± 0.52	0.361	< 1.47
HDL‐C (mmol/L)	1.26 ± 0.04	1.49 ± 0.01	1.31 ± 0.03	0.050	≥ 1.04
LDL‐C (mmol/L)	2.16 ± 0.40	2.83 ± 0.29	2.59 ± 0.38	0.299	< 3.35
LS aBMD (g/cm^2^)	0.672 ± 0.08	0.646 ± 0.10	0.629 ± 0.10	0.452	
LS aBMD *Z*‐score	0.128 ± 1.28	0.194 ± 1.11	0.246 ± 1.33	0.965	≥ −2.0
FN aBMD (g/cm^2^)	0.589 ± 0.10	0.582 ± 0.12	0.597 ± 0.08	0.935	
FN aBMD *Z*‐score	−2.288 ± 1.59	−1.960 ± 1.55	−1.62 ± 1.29	0.484	≥ −2.0
TR aBMD (g/cm^2^)	0.380 ± 0.09	0.400 ± 0.11	0.410 ± 0.08	0.673	
TH aBMD (g/cm^2^)	0.549 ± 0.10	0.563 ± 0.11	0.596 ± 0.07	0.392	
TBS	1.328 ± 0.10	1.326 ± 0.12	1.295 ± 0.12	0.621	
TBS *Z* score	−1.162 ± 0.97	−0.673 ± 0.85	−0.646 ± 0.70	0.327	

*Note:* Bold values indicate that there was a signification difference among 3 groups.

Abbreviations: 25(OH)D, 25‐hydroxyvitamin D; ALN, alendronate; ALP, alkaline phosphatase; ALT, alanine aminotransferase; aBMD, areal bone mineral density; BMI, body mass index; \(\beta \)‐CTX, \(\beta \)‐isomerized carboxy‐telopeptide of type I collagen; Ca, calcium; Cr, creatinine; DMD, Duchenne muscular dystrophy; FBG, fasting blood glucose; FN, femoral neck; GC, glucocorticoid; HDL‐C, high‐density lipoprotein cholesterol; IQR, interquartile range; LDL‐C, low‐density lipoprotein cholesterol; LS, lumbar spine; P, phosphate; PTH, parathyroid hormone; SD, standard deviation; TBS, trabecular bone score; TC, total cholesterol; TG, triglycerides; TH, total hip; TR, trochanter; VCF, vertebral compression fracture; ZOL, zoledronic acid.

* *p* < 0.05 vs. control group.

** *p* < 0.01 vs. control group.

**FIGURE 1:**
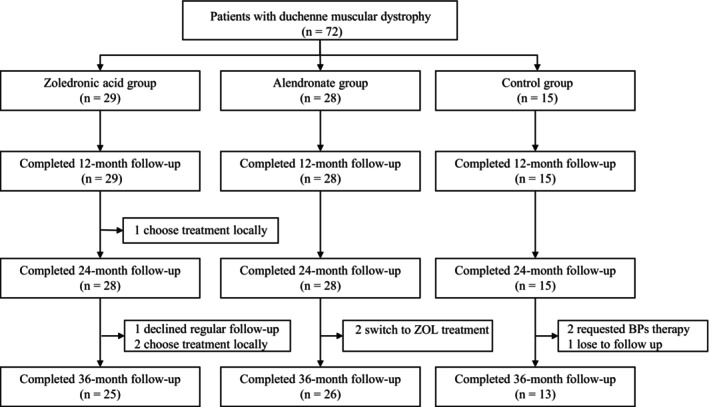
Flow chart of the study.

Sixty‐four patients (88.9%) completed the 36‐month follow‐up (Figure 1). In the ZOL group, three patients were referred to a local hospital for treatment and subsequently lost to follow‐up and another patient was lost to follow‐up for unknown reasons. In the alendronate group, two patients discontinued treatment prematurely and switched to other therapies. In the control group, one patient requested to withdraw and switched to alendronate treatment, while another was lost to follow‐up for unknown reasons.

### 3.2 Changes in TBS and TBS Z‐Score

**FIGURE 2:**
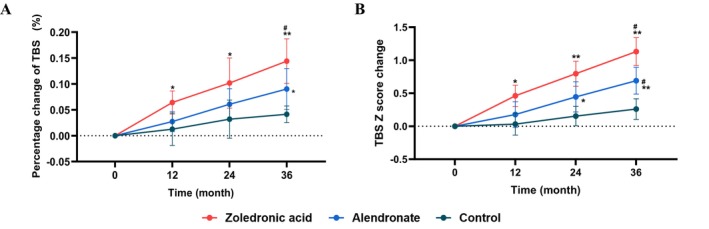
Changes in lumbar spine trabecular bone score (TBS) and TBS Z‐score during treatment. A. Percentage change in TBS during the treatment. B. Changes in TBS Z‐score during the treatment. Both the zoledronic acid and alendronate groups showed significant increases in TBS and TBS Z‐score, with no statistically significant difference observed between the two groups. Abbreviations: TBS: trabecular bone score. Data are shown as the mean and standard error.. *p < 0.05, **p < 0.01, ***p < 0.001 vs. baseline. #: *p* < 0.05, ##: *p* < 0.01, ###: *p* < 0.001 vs. control group.

The baseline TBS values of patients in the ZOL group, alendronate group and control group were 1.328 ± 0.10, 1.326 ± 0.12 and 1.295 ± 0.12, respectively, with TBS Z‐scores of −1.162 ± 0.97, −0.673 ± 0.85 and −0.646 ± 0.70, respectively. No statistically significant differences were observed in TBS and its Z‐score among the three groups. After 3 years of treatment, TBS increased by 14.3%, 9.2% and 4.1% in the ZOL group, alendronate group and control group (*p* < 0.01, *p* < 0.05, *p* > 0.05 vs. baseline), respectively. The increase in TBS in the ZOL group was significantly higher than that in the control group (*p* < 0.05), but did not differ significantly from the increase in the alendronate group (Figure 2A). Similarly, the TBS Z‐score increased by 1.13 (*p* < 0.01 vs. baseline), 0.68 (*p* < 0.01 vs. baseline) and 0.26 (*p* > 0.05 vs. baseline) in the ZOL group, alendronate group and control group (Figure 2B). There was a significant difference between the two bisphosphonate treatment groups and the control group (all *p* < 0.05), while no significant difference was found between the ZOL and alendronate groups.

### 3.3 Changes in aBMD and aBMD Z‐Score

**FIGURE 3:**
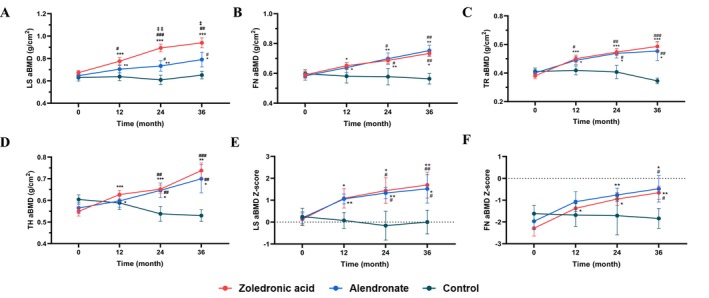
Changes in areal bone mineral density (aBMD) and aBMD Z‐score during treatment. (A) Changes in aBMD at the lumbar spine during treatment. (B) Changes in aBMD at the femoral neck during treatment. (C) Changes in aBMD at the trochanter during treatment. (D) Changes in aBMD at the total hip during treatment. (E) Changes in lumbar spine aBMD Z‐score during treatment. (F) Changes in femoral neck aBMD Z‐score during treatment. Compared with the control group, both the zoledronic acid and alendronate groups showed significant increases in aBMD and aBMD Z‐scores at the lumbar spine and hip. Moreover, the increase in lumbar spine aBMD was greater with zoledronic acid than with alendronate. Abbreviations: LS: lumbar spine, FN: femoral neck, TR: trochanter, TH: total hip, aBMD: areal bone mineral density. Data are shown as the mean and standard error. *: *p* < 0.05, **: *p* < 0.01, ***: *p* < 0.001 vs. baseline. #: *p* < 0.05, ##: *p* < 0.01, ###: *p* < 0.001 vs. control group. ‡: *p* < 0.05, ‡‡: *p* < 0.01 between the zoledronic acid group and alendronate group.

After 3 years of treatment, patients' aBMD at LS, FN, TR and TH significantly increased by 35.8%, 23.7%, 55.3% and 34.5% (*p* < 0.001 or *p* < 0.01 vs. baseline) in the ZOL group and by 21.5%, 29.3%, 37.5% and 25.0% in the alendronate group (all *p* < 0.05 vs. baseline) (Figure 3). The control group displayed changes in aBMD at LS, FN, TR and TH by 3.1%, −3.0%, −12.2% and −11.7% (all *p* > 0.05 vs. baseline). The increases in LS aBMD in the ZOL group were significantly higher than those in the alendronate group (*p* < 0.05), while the changes in aBMD at other sites did not differ between the two groups (*p* > 0.05). Besides, LS and FN aBMD Z‐score also increased by 1.56 and 1.63 in the ZOL group (all *p* < 0.01 vs. baseline) and by 1.32 and 1.48 in the alendronate group (all *p* < 0.05 vs. baseline). There was no significant difference in aBMD Z‐scores at various sites between the ZOL and alendronate groups. The increases in aBMD and aBMD Z‐score in the ZOL and alendronate groups were significantly higher than those in the control group (all *p* < 0.05 vs. control group) (Figure 3).

### 3.4 Changes in Serum Biochemical Indicators

**FIGURE 4:**
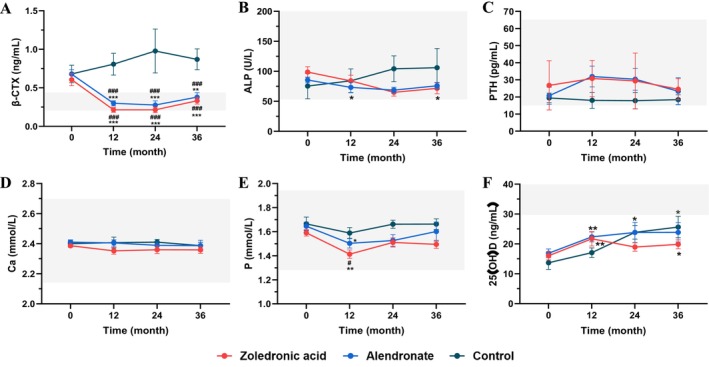
Changes in bone metabolic markers during treatment. (A) Changes in serum concentrations of β‐isomerized carboxy‐telopeptide of type I collagen. (B) Changes in serum concentrations of alkaline phosphatase. (C) Changes in serum concentrations of parathyroid hormone. (D) Changes in serum calcium concentrations. (E) Changes in serum phosphate concentrations. (F) Changes in serum concentrations of 25‐hydroxyvitamin D. Both the zoledronic acid and alendronate treatment groups demonstrated a significant reduction in serum β‐isomerized carboxy‐telopeptide of type I collagen (a bone resorption marker). Abbreviations: β‐CTX: β‐isomerized carboxy‐telopeptide of type I collagen (a bone resorption marker), ALP: alkaline phosphatase (a bone formation marker), PTH: parathyroid hormone, Ca: calcium, P: phosphate, 25(OH)D: 25‐hydroxyvitamin D. Data are shown as the mean and standard error. *: *p* < 0.05, **: *p* < 0.01, ***: *p* < 0.001 vs. baseline. #: *p* < 0.05, ##: *p* < 0.01, ###: *p* < 0.001 vs. control group.

After 3 years of treatment, serum β‐CTX levels decreased significantly from baseline by 45.9% and 44.1% in the ZOL group (*p* < 0.001) and alendronate group (*p* < 0.01), but increased by 28.0% in the control group (*p* > 0.05 vs. baseline). There was no significant difference in the changes in β‐CTX levels between the ZOL and alendronate groups (Figure 4A). The serum ALP levels in the ZOL and alendronate groups decreased by 27.5% (*p* < 0.05 vs. baseline) and 14.7% (*p* > 0.05 vs. baseline) and increased by 29.2% (*p* > 0.05 vs. baseline) in the control group (Figure 4B).

No significant differences in serum levels of PTH and calcium were found among the three groups during the three‐year follow‐up (Figure 4C,D). After 12 months of treatment with ZOL and alendronate, serum P levels significantly decreased from baseline (*p* < 0.01 and *p* < 0.05, respectively). Importantly, these post‐treatment levels remained within the normal physiological range. During the subsequent follow‐up, serum P levels gradually returned to baseline levels (Figure 4E). During the 36‐month follow‐up, serum 25(OH)D levels in all three groups showed an increasing trend but without significant differences from baseline (Figure 4F).

### 3.5 Safety of the Treatment

During the 3‐year treatment period, no serious AEs were observed. No renal impairment, hypocalcemia, severe infections, osteonecrosis of the jaw, atypical fractures, atrial fibrillation or deaths were found in the three groups (Table 2).

**TABLE 2:** Adverse events during the treatment.

Adverse events	ZOL group (*n* = 29)	ALN group (*n* = 28)	Control group (*n* = 15)	*p*
Headache, *n* (%)	1 (3.4%)	0 (0)	0 (0)	0.570
Fever, *n* (%)	22 (75.9%) ***	0 (0)	0 (0)	< 0.001
Nausea, *n* (%)	0 (0)	6 (21.4%) *	0 (0)	0.014
Vomiting, *n* (%)	0 (0)	0 (0)	0 (0)	/
Abdominal pain, *n* (%)	0 (0)	0 (0)	0 (0)	/
Myalgia, *n* (%)	3 (10.3%)	2 (7.1%)	0 (0)	0.731
Bone pain, *n* (%)	4 (13.8%)	3 (10.7%)	0 (0)	0.607
Rash, *n* (%)	0 (0)	0 (0)	0 (0)	/
Renal dysfunction, *n* (%)	0 (0)	0 (0)	0 (0)	/
Hypocalcemia, *n* (%)	0 (0)	0 (0)	0 (0)	/
Hypercalcemia, *n* (%)	0 (0)	0 (0)	0 (0)	/
Atypical fracture, *n* (%)	0 (0)	0 (0)	0 (0)	/
Osteonecrosis of the jaw, *n* (%)	0 (0)	0 (0)	0 (0)	/
Atrial fibrillation, *n* (%)	0 (0)	0 (0)	0 (0)	/
Death, *n* (%)	0 (0)	0 (0)	0 (0)	/

*Note:* The bold indicates that there is a statistical difference between the ZOL group and the ALN group. Values are given as number (proportion).

Abbreviations: ALN: alendronate, ZOL: zoledronic acid.

* *p* < 0.05.

*** *p* < 0.001 vs. the control group.

AEs were found in 23 patients (79.3%) and 8 patients (28.6%) in the ZOL group and alendronate group, respectively (Table 2). The AEs primarily included gastrointestinal adverse reactions such as nausea, acute‐phase reactions such as fever, bone pain and myalgia. Acute‐phase reactions, such as fever, were more frequent in the ZOL group than in the alendronate group (75.9% vs. 0%, *p* < 0.001). Gastrointestinal adverse reactions, such as nausea, were more frequent in the alendronate group than in the ZOL group (21.4% vs. 0%, *p* < 0.01). Serum ALT levels in all three groups were higher than the normal range but similar to the baseline. Serum Cr levels were significantly lower than the normal range in DMD patients, with no differences among the three groups. Serum FBG levels were within the normal range among the three groups (Figure S1).

## 4 Discussion

DMD is a rare, X‐linked recessive and severely debilitating disease characterized by progressive muscle degeneration and is frequently complicated by osteoporosis and fragility fractures [20]. GCs are currently the mainstay of recommended treatment for DMD patients, as they are beneficial to improve muscle strength [1]. However, long‐term use of GCs can promote osteoclast formation, reduce osteoblast activity, promote the breakdown of bone matrix proteins, induce osteoporosis and fragility fractures, which can accelerate the loss of ambulation and impair cardiovascular and pulmonary function [21]. In addition, DMD patients have significantly decreased muscle strength, resulting in reduced mechanical stimuli on the bones, which not only reduces bone formation but also increases the risk of falls [22]. In this study, 23.6% of children with DMD had a history of fragility fractures, even 27.8% had lost independent ambulation, and both TBS Z‐score of LS and aBMD Z‐score of FN were lower than sex‐ and age‐matched paediatric reference levels. Therefore, focusing on and treating bone abnormalities in DMD patients holds significant clinical importance.

This three‐year prospective study demonstrated that bisphosphonate treatment—whether oral or intravenous formulations—could effectively improve TBS Z‐scores in DMD patients with osteoporosis. This study is the first to utilize TBS to assess bone microstructure in DMD patients with osteoporosis and to evaluate the effects of various bisphosphonates on TBS, thereby filling a critical gap in bone microstructure evaluation for this disease. These findings support the use of TBS as an image parameter to reflect bone microstructure and efficacy of anti‐osteoporosis treatment in DMD patients.

As we know, bisphosphonates are synthetic analogs of naturally occurring pyrophosphates and can increase aBMD in patients with GIOP and primary osteoporosis by reducing osteoclast activity [23]. Our previous study demonstrated that alendronate and ZOL therapy for 2 years could effectively increase aBMD in children with DMD [24]. A meta‐analysis has shown that bisphosphonates effectively increased the LS aBMD Z‐score in patients with DMD and GIOP [12]. This 3‐year prospective study not only expands the subject cohort and extends the treatment duration but also evaluates the improvement of bone microstructure through TBS, thereby providing a comprehensive assessment of the skeletal benefits of bisphosphonates in DMD patients with osteoporosis.

Notably, bone strength is determined by both aBMD and, equally critically, microarchitectural properties [25]. Previous research indicated that long‐term GC treatment in DMD patients leads to GIOP, causing significant deterioration of bone microstructure and markedly increased fracture risk independent of aBMD [26]. In fact, after receiving GC treatment, fractures can occur even when aBMD is not severely low [27]. TBS is a novel grey‐level texture measurement acquired from DXA aBMD images that provides an indirect assessment of bone microarchitecture [28]. Compared to traditional aBMD, which only measures bone mass, TBS analyses the texture and connectivity of trabecular bone, providing additional information on bone strength and compensating for the limitations of aBMD. Thus, TBS can be used to assess bone microstructure and predict fracture risk, adding useful information for treatment decision‐making and monitoring [28]. Our previous study evaluated TBS for assessing vertebral fractures and spinal deformity in children and adolescents with osteogenesis imperfecta and proved that TBS showed superior performance in identifying vertebral fractures compared to aBMD, especially in patients without densitometric osteoporosis, suggesting its potential for monitoring vertebral fractures and spinal deformity risk [29]. In this study, we first employed TBS to evaluate bone microstructural integrity in DMD patients with osteoporosis. Furthermore, we provide pioneering evidence on the therapeutic efficacy of bisphosphonate (both oral and intravenous formulations) in improving TBS Z‐scores in DMD‐associated osteoporosis. Moreover, TBS may serve as a complementary tool for prediction of fracture risk and for evaluation of treatment efficacy in DMD patients with osteoporosis.

We evaluated the safety of long‐term treatment of ZOL and alendronate for osteoporosis in children and adolescents with DMD. The overall safety of alendronate and ZOL was generally well‐tolerated. However, acute‐phase reactions were significantly more common after the initial zoledronic acid infusion than with alendronate. These reactions were effectively alleviated with non‐steroidal anti‐inflammatory drugs. No serious AEs were observed in this study, which is consistent with previous reports in DMD populations [30]. In addition, it is worth noting that serum ALT levels were significantly higher than the normal range in all patients with DMD, whereas serum Cr levels were significantly lower than the normal range at baseline. As DMD is a progressive muscle disease caused by the absence of dystrophin and leading to muscle atrophy and degeneration, muscle mass is significantly reduced [4]. The loss of muscle cell membrane stability leads to continuous muscle fibre necrosis, resulting in the release of intracellular components including ALT into the bloodstream [31]. Since Cr is a byproduct of muscle metabolism, the loss of muscle mass directly reduces Cr production. Additionally, the limited mobility of DMD children further reduces Cr generation [32].

This study is the first to employ TBS for evaluating bone microstructure in children and adolescents with DMD and confirmed that treatment with ZOL and alendronate not only significantly improved the aBMD of DMD patients but also enhanced the bone microstructure, as evidenced by increased TBS values. The study has accumulated valuable clinical experience for the treatment of osteoporosis in children and adolescents with DMD. However, there are still a series of limitations in this study. The dosage of GCs was individualized by the neurologists. We did not rule out the influence of different GCs doses on the results. The study did not employ validated functional assessment scales such as the NSAA or PUL to evaluate muscle strength and activity ability. Consequently, it cannot determine the impact of bisphosphonates on motor function in patients with DMD. Future prospective studies should incorporate validated functional assessments to comprehensively evaluate the clinical impact of bisphosphonate therapy in patients with DMD. The sample size was not large enough and the study period was not long enough to assess the impact of bisphosphonate treatment on bone fracture risk. Additionally, no assessment was performed regarding the correlation between genotype and osteoporosis severity response to bisphosphonate therapy.

While DMD remains incurable, significant advances have been made in disease‐modifying therapies that delay disease progression and improve quality of life [33, 34]. Promising strategies encompass genetic modulation approaches, such as exon‐skipping antisense oligonucleotides and gene transfer/editing techniques [35]. The former induces the exclusion of specific mutant exons to enable production of truncated but functional dystrophin [36, 37]. Gene supplementation strategies, including adeno‐associated virus (AAV)‐mediated delivery of micro‐dystrophin transgenes, aim to provide a functional gene copy. Separately, gene editing technologies like CRISPR/Cas9 are being developed to correct the mutation directly [38, 39, 40]. As these therapies targeting the primary genetic defect advance, supportive interventions—such as bone health management—remain essential [41]. Our findings demonstrate that bisphosphonates effectively improve bone microarchitecture and BMD, thereby strengthening the supportive care framework for managing secondary osteoporosis in DMD.

## 5 Conclusion

Patients with DMD face multiple threats to bone health due to progressive muscle weakness, restricted mobility and long‐term GCs therapy, ultimately leading to fragility fractures and a progressive decline in quality of life. By inhibiting osteoclast‐mediated bone resorption, bisphosphonates—whether the intravenous agent ZOL or the oral agent alendronate—effectively improve bone microarchitecture and bone strength in children and adolescents with DMD, as evidenced by increased TBS and aBMD. This population demonstrates good overall tolerance to both ZOL and alendronate. TBS may serve as a supplementary imaging indicator for evaluating the efficacy of bisphosphonate therapy for osteoporosis in patients with DMD.

## Funding

This work is supported by National High Level Hospital Clinical Research Funding (2022‐PUMCH‐B‐014) and the CAMS Innovation Fund for Medical Sciences (CIFMS) (2021‐I2M‐1‐051).

## Ethics Statement

The study complied with the ethical guidelines for authorship and publishing set by the *Journal of Cachexia*, *Sarcopenia and Muscle*.

## Conflicts of Interest

The authors declare no conflicts of interest.

## Supporting information


**Figure S1:** Changes in biochemical markers during treatment.
