# Supplementary material for: Effects of Bisphosphonates on Bone Micro‐Architecture of Children With Duchenne Muscular Dystrophy: A Prospective Comparative Study
Source: J Cachexia Sarcopenia Muscle. 2026 Feb 26;17(2):e70227. doi: 10.1002/jcsm.70227 (PMC12945921; doi:10.1002/jcsm.70227)
Supplement: Supplementary file 1 — Figure S1: Changes in biochemical markers during treatment. [file JCSM-17-e70227-s001.pptx]

## Slide 1
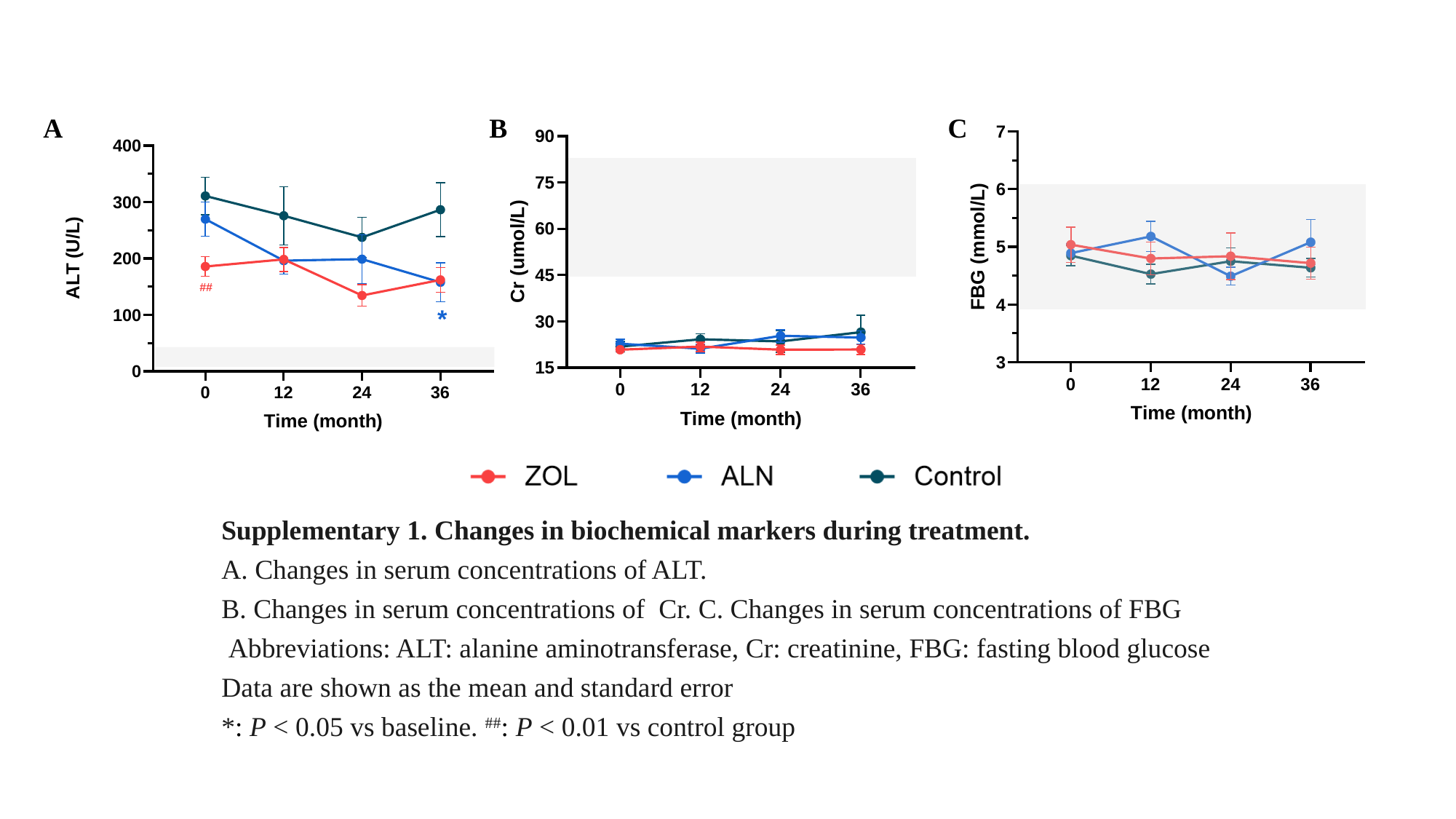

A
B
C
Supplementary 1. Changes in biochemical markers during treatment.
A. Changes in serum concentrations of ALT.
B. Changes in serum concentrations of Cr. C. Changes in serum concentrations of FBG
 Abbreviations: ALT: alanine aminotransferase, Cr: creatinine, FBG: fasting blood glucose
Data are shown as the mean and standard error
*: P < 0.05 vs baseline. ##: P < 0.01 vs control group
